# Guideline for feedback of individual genetic research findings for genomics research in Africa

**DOI:** 10.1136/bmjgh-2021-007184

**Published:** 2022-01-10

**Authors:** Alice Matimba, Stuart Ali, Katherine Littler, Ebony Madden, Patricia Marshall, Sheryl McCurdy, Victoria Nembaware, Laura Rodriguez, Janet Seeley, Paulina Tindana, Aminu Yakubu, Jantina de Vries, the H3Africa Ethics and Community Engagement Working Group

**Affiliations:** 1 Wellcome Connecting Science, Wellcome Genome Campus, Hinxton, UK; 2 Akili Labs (Pty) Ltd, Johannesburg, South Africa; 3 Health Ethics & Governance Unit, World Health Organization, Geneve, Switzerland; 4 National Human Genome Research Institute, NIH, Bethesda, Maryland, USA; 5 Department of Bioethics, School of Medicine, Case Western Reserve University, Cleveland, Ohio, USA; 6 Center for Health Promotion and Prevention Research, The University of Texas Health Science Center at Houston, Houston, Texas, USA; 7 Division of Human Genetics, Deparment of Pathology, University of Cape Town, Rondebosch, Western Cape, South Africa; 8 Department of Global Health & Development, London School of Hygiene and Tropical Medicine, London, UK; 9 School of Public Health, University of Ghana, Legon, Greater Accra, Ghana; 10 Center for Bioethics and Research, Ibadan, Oyo, Nigeria; 11 National Health Research Ethics Committee, Federal Ministry of Health, Nigeria, Nigeria; 12 54gene, Nigeria, Nigeria; 13 Department of Medicine, University of Cape Town, Rondebosch, Western Cape, South Africa

**Keywords:** health policy, health services research

## Abstract

As human genomics research in Africa continues to generate large amounts of data, ethical issues arise regarding how actionable genetic information is shared with research participants. The Human Heredity and Health in Africa Consortium (H3Africa) Ethics and Community Engagement Working group acknowledged the need for such guidance, identified key issues and principles relevant to genomics research in Africa and developed a practical guideline for consideration of feeding back individual genetic results of health importance in African research projects. This included a decision flowchart, providing a logical framework to assist in decision-making and planning for human genomics research projects. Although presented in the context of the H3Africa Consortium, we believe the principles described, and the decision flowchart presented here is applicable more broadly in African genomics research.

**Summary points**

- Over the last decade, there has been an increased generation of human genomic data and a growing need to develop guidance for feeding back actionable genomic research findings from African research projects.
- We outline key issues and principles relevant to genomics research in Africa and provide a practical decision tree approach for deciding what, and when, to feedback.
- We recommend that researchers include plans for feedback of actionable genomic research findings to research participants at the project proposal stage, obtain appropriate informed consent, make sure resources are allocated and ensure sufficient capacity and expertise is available to effectively support the feedback process.
- Our guideline recommends tailoring implementation, taking into consideration contextual factors and heterogeneity of African settings.

## Introduction

The debate about whether and how individual genetic research findings should be returned to research participants persists, with little guidance available for how this should be done particularly in the African settings.^1 2^ Research to determine preferences and perspectives of African stakeholders on the feedback of individual genetic results to participants in genomics research projects (feedback of findings; FoFs) is ongoing.^3 4^ This supports the call to action for understanding contextual factors in African communities that impact on decision-making regarding FoFs and guides development of national and regional guidelines. Globally, the subject of return of genetic results including incidental findings has gained momentum in favour of disclosure of clinically actionable genomic findings from research studies in an ethically and legally appropriate manner. Few guidelines have been developed, which outline procedures for the feedback process and could provide frameworks for adapting to research contexts and jurisdictions.^5–9^ Recently, the African Academy of Sciences (AAS) published a policy paper, which includes recommendations for integration of genomic medicine research and service delivery^10^ and can be used to extrapolate actionability strategies alongside this guideline.

**Figure 1:**
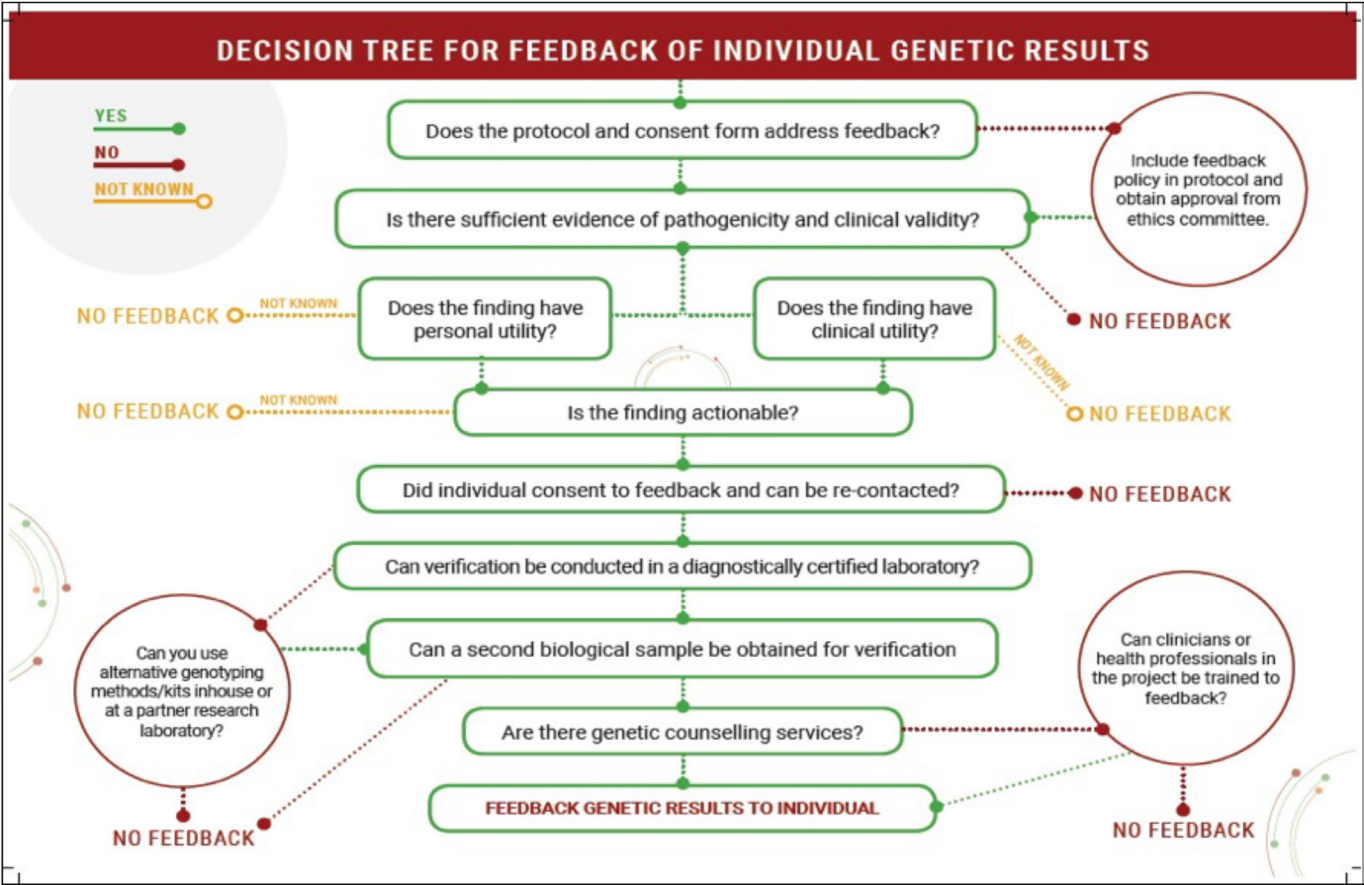
Decision flowchart for feedback of individual genetic results.

The Human Heredity and Health in Africa (H3Africa) Initiative brought together researchers to build capacity and study genomics and environmental determinants of common diseases of relevance to African populations.^11–13^ Supporting genomics research in 34 countries, H3Africa projects promoted application of genomics technologies, infrastructure development, training and ethics aimed at understanding the role of population diversity in health and disease. Many research projects collect samples from participants and generate large amounts of genomic data, thereby raising ethical challenges. To date, H3Africa supported development and implementation of various ethics and governance frameworks for genomics research.^14–16^ Having identified an urgent need to develop guidelines for returning individual genetic research findings, the H3Africa Ethics and Community Engagement Working group outlined key issues and principles relevant to genomics research in Africa and provided a guided practical approach when considering FoFs in African research projects. A decision flowchart (figure 1), which provides a logical framework to assist in planning and decision-making on whether or not to provide FoFs, is also presented for further practical guidance.

### Guideline development

In 2016, a taskforce consisting of members of the H3A Ethics and Community Engagement working group was set up to develop guidance, which takes into account contextual factors when considering FoFs in research projects based in Africa. A consultation workshop and a series of online meetings were held to understand the unique issues and challenges of feeding back genetic results from studies conducted in Africa. This was instrumental in mapping out key contextual features, which were central to the task force deliberations. These included (1) varying degrees of resource limitations, expertise and capacity for genomics, (2) absence, or lack of implementation, of relevant guidelines in genomics research, (3) diverse socioeconomic and cultural environments, (4) differing health systems and delivery capabilities. The task force also acknowledged the heterogeneity of African settings and research projects and aimed to provide generalisable guidance for African researchers who planned for FoFs at the start of their projects.

The decision tree (figure 1) was developed as a summary guide for researchers, providing a step-by-step checklist. By first applying the general principles—participant volition, utility and validity, we used a logical approach to order the steps into a decision tree starting with having a policy for research networks, an ethically approved informed consent framework and clear strategy for feeding back actionable genetic results to research participants. For each step, considerations were made on practicality, potential challenges and possible solutions as further detailed in the guideline text. Using the decision tree at the start of a research project would enable researchers to decide on feasibility of FoFs, identify areas of resource needs and scope of work. The guideline and decision tree were reviewed by H3Africa Consortium researchers involved in large-scale genomics studies based in multiple countries in Africa.

### A guideline for feedback of individual genetic research findings for genomics research in Africa

The H3Africa Ethics and Community Engagement Working Group developed a set of guiding principles to inform decisions about returning individual genetic research findings to research participants by the H3Africa Consortium members, which is broadly applicable to genomics research in Africa. As a preamble to the guiding principles, we highlight two important considerations—what are the generally agreed criteria that need to be met before considering feeding back results to participants and what are the situations under which feedback ought not to be considered?

#### General consensus for FoFs

Decisions about whether and what findings ought to be fed back should be based on analytical and clinical validity of the results, their potential value and utility as well as participant volition.^2^ Therefore, to consider feedback, it is recommended that the following criteria to be met.

1. Methods used to generate those findings should be able to accurately and reliably detect genetic variant(s) in the affected individuals (high analytical validity).
2. Genetic variant(s) should be strongly associated with disease causation and be able to accurately and reliably predict clinical outcome (ie, high clinical validity).
3. Findings should be able to guide therapy, life choices or prevent disease (clinical utility) AND/OR have proven therapeutic or preventive intervention (medical actionability).^2^ There should also be indication that participants wish to receive findings that fulfil these criteria, preferably following a process addressed during study consent or enrolment.^17^
4. Participants should also be afforded the right not to know.^18^

#### Exclusions and exceptions for FoF consideration

- This guideline expressly excludes projects that recruit participants in distant locations on a one-off basis, without any ongoing relations between the research team and the research participants.
- FoFs should be limited to the primary research study and exclude secondary use of samples and data analysis.
- In addition to revealing pertinent findings related to the condition under investigation, incidental findings may be found. It is advisable to prioritise study-related results that are pertinent to clinical diagnosis or treatment; and that only validated incidental findings be considered where appropriate (see the Feedback of incidental findings section below).

## Guiding principles

In the face of uncertainty about whether and which results should be fed back, most H3Africa researchers were not supportive of feeding back individual genetic research results.^19^ With increased understanding, however, H3Africa recognised that there were cases where FoFs could be important for the health and well-being of research participants and their families. Essentially, FoFs should be deeply considered when the associated risk for the disease is significant; there are important health implications such as premature death or substantial morbidity; there are significant reproductive implications or proven therapeutic or preventive interventions are available.^20 21^ However, a decision to feed back findings also needs to consider all of the items discussed below.

### Primary conditions in deciding to feedback individual genetic results

We recommend the following key considerations when making a decision for FoFs.

#### Primarily feedback findings that relate to the disease being investigated in the research project

Where researchers decide to provide FoFs, they should focus primarily on feeding back pertinent findings, which are related to the disease or condition that is being investigated, and in which the research team has both clinical and analytical expertise. In that case, we assume that the research teams would be:

- in a position to review and assess the evidence base for potentially pathogenic variants in relation to the population(s) that are being investigated.
- able to assess whether the particular finding likely holds value for the individual.
- able to verify the finding(s) using an analytically validated assay and repeat the test with another sample collected from the same patient/subject and/or test in a certified diagnostic laboratory (see the If we do decide to feedback, how should it be done? section below).
- able to ensure that patients are appropriately informed of the implications of the findings for their disease and/or treatment.
- able to advise on, and refer to, appropriate follow-up care.

Where these conditions are met in the study design, researchers should consider whether their research is likely to identify findings that should be fed back and consult with the funders and health service providers on how this could be supported. It is noted that in only a few of the H3A projects, these criteria were adequately fulfilled to consider FoFs. However, the research team should determine the nature of results to be fed back and the steps to be taken, in consultation with Institutional Review Boards or Research Ethics Committees (IRB/RECs) and other relevant stakeholders.

#### Role of research ethics committees in FoFs

We recommend that investigators should include provisions on feeding back findings in their consent forms and should consult with and receive approval from the local REC that approved the research project. Specifically:

- If feedback is anticipated, the plan to do so should be clearly stated in the initial protocol submission.
- Alternatively, if feedback becomes desirable subsequent to approval of the study protocol, the plan to provide feedback should be clearly stated in a supplementary protocol or study amendment.
- The FoF plan should be reviewed and approved as part of the design of the research project and included in an appropriate way within the informed consent process and project proposal documents.
- In the case of children, there is an additional responsibility to ensure that experimental protocols are aligned with respective local statutes and international regulations/declarations that protect the rights of minors.

It is recommended that IRB/RECs in Africa (and elsewhere) draw on specialised expertise such as medical geneticists, clinicians, genetic counsellors, ethicists, bioinformaticians and in consultation with the community advisory board, where these exist if relevant expertise does not exist within the board/committee when making these assessments.^8 21^ The purpose of such a committee would be to generate guidance on what is reportable in genetic studies, provide for broad stakeholder input, allow a more consistent approach across research networks or consortia and to provide credible guidance for the researchers and IRBs.^21^

#### Time frame

Since research projects are time and resource limited, it may be necessary to provide a time frame, indicating how long it may be before participants can expect any individual study results to be shared. In this case, it is advisable to assess practicalities of FoFs before the start of the project in order to ensure that the time and capacity are well appropriated. For monogenic disease studies, findings with potential medically actionable findings should be provided as soon as results are validated. There are many uncertainties surrounding the appropriateness of returning individual research results, and it may be difficult to trace individual research participant years after recruitment. Therefore, we strongly suggest that the return of study results should be limited to the primary research study and not to secondary use of samples and data.

### Pathogenicity and clinical validity of research findings

Suggested variants that are potentially appropriate for reporting back findings should always be reviewed by investigators from individual studies for appropriateness of reporting in their study. Evidence of pathogenicity can be determined from various sources such as population genomic epidemiology data, computational predictive methods, functional studies or segregation data. Some variants may be predicted to be pathogenic but not actionable (ie, no medical interventions exist that would avert the illness). Therefore, regardless of the analytical methods applied, clinical validity should be ascertained, whereby there is both sufficient evidence that the variant or related gene/protein results in the target phenotype or disease symptoms, and that it is relevant for the target population.

Challenges in clinical validation include phenotypic heterogeneity, pleiotropy (single genetic loci affecting multiple phenotypes), incomplete penetrance, confounding of phenotypic modifiers (eg, environment, lifestyle) and a limited scientific evidence base.^20 22^ This is less problematic for monogenic disorders or for complex traits, whereby variants have large effect sizes. While most H3Africa projects work on common complex diseases with multifactorial causes, a few studies are focused on monogenic disorders. In providing FoFs for the latter, and to some extent, highly heritable complex diseases, it may be easier to determine pathogenicity and clinical validity because of well-established phenotype–genotype link for known genes, and in rare cases, novel genetic markers, with very strong evidence for causality. Most other complex diseases comprise weaker genetic associations spread over a large number of genomic loci.^23^ Population-validated genomic risk profiles may be established based on ongoing research in Africa. These challenges for determining pathogenicity and clinical validity are ever more pronounced in populations of African ancestry and Africa as a whole as a result of the higher levels of genetic diversity on the African continent.^24 25^ To strengthen this evidence base, H3Africa investigators may be well placed to contribute to initiatives such as the Clinical Genome Resource (ClinGen) (https://clinicalgenome.org) and in future to develop guidelines to set the evidence threshold, which can be used for determining pathogenicity and clinical validity.

### Establishing value

One key component of discussions about FoFs relates to questions about whether receiving individual results is likely to be of value to the participant. There are two ways in which individual results can be of value: either because they have clinical utility and/or are medically actionable, meaning that information could be used to guide diagnosis and treatment and there is some medical intervention available that would improve outcomes for patients,^17 18^ or because they have ‘personal utility’, which includes considerations of how participants would use research findings confirmed in a diagnostic laboratory, including, for instance, a genetic diagnosis that ends a diagnostic odyssey to understand a life-long medical condition or reveals a carrier status that could inform reproductive decisions. This excludes cases of confirming identity and paternity and other forensic uses. Therefore, clinical utility, medical actionability and personal utility—are good reasons to feedback individual findings in genomics research. Thus, when deciding to feedback findings, researchers need to explain how results will likely be of value to individuals receiving them.

#### Is there evidence of clinical utility?

When genetic testing strongly predicts an adverse clinical outcome, it is of high clinical utility, providing that there is guidance on a possible intervention. While the effects of pathogenic variants in monogenic disease cases are relatively significant, complex diseases often present several genetic variants, which individually have lower predictive values and lower clinical utility as disease risk predictors.^26 27^ In addition, there are often no treatments or preventive measures that substantially reduce risk; therefore, relevant genetic results with clinical utility in complex diseases are uncommon. Programmes such as ClinGen and ClinVar use combinations of evidence from clinical and public health data, basic science and in silico research to identify and curate genetic variants with clinical utility.^28 29^ Engelbrehct *et al* demonstrated clinical utility of sequencing panels in improving management of primary immunodeficiency disorders in an African setting.^30^ It is important to note that clinical utility does not guarantee actionability, especially in Africa, where limited access to resources could limit provision of appropriate care. However, the information may be useful in understanding diagnosis or making reproductive decisions. Although little progress has been made regarding clinical utility for complex traits, genetic markers of pharmacological response may be useful in advising drug treatment or alerting an individual of a drug toxicity risk.^10^ For diseases such as cancer, genetic risk scores and other genetic markers may be applicable for preventive, predictive and prognostic use.

#### Actionability: adopt one standard for the entire project

One critical feature of these discussions is that, to date, what counts as medically actionable has not taken into account the resources available in the setting where participants are based, and the treatment options available to them.^31^ For example, what may be actionable in one country or healthcare setting may not be actionable for someone else even if they reside in the same region or country due to socioeconomic and other factors. Emerging consensus in the H3Africa Consortium is that it would not be appropriate to adopt different feedback policies for people in different collaborating sites in the same project. This could be paternalistic and unfairly prevent participants from knowing something because they do not have the resources to act on the information. Also, even if a particular intervention is not available in the public healthcare system in a country, researchers are not in a position to know whether participants have recourse to other means of acting on results. For instance, people may have family members abroad who could pay for interventions, or they may have access to non-governmental organisations that offer healthcare. It is common for medical professionals in under-resourced healthcare settings to inform their patients of what care they can receive in the public healthcare system and what care they could receive if they could pay for it. The implication is that where a given project decides to feedback pertinent reportable individual genetic finding(s), it should include all participants in that project, regardless of whether or not those findings are actionable in their particular contexts. Researchers should be mindful that treatment options may change in the future, and, therefore, when deciding on feedback, they should assess any ongoing moral obligations to reassess treatment options for participants who have received FoFs. Engagement with relevant RECs is required in order to ensure that they are familiar with the research trends and the potential benefits for participants and guide decisions across consortia study sites.

### Volition

#### Include options for feedback in the consent process

Internationally, one key criterion determining whether individual genetic research results should be fed back is the preference expressed by the participant to receive such results.^2^ While this would normally be addressed in the informed consent document, there is ample evidence suggesting that consent processes for genomics research in Africa are already overburdened, and that participant comprehension is low in most studies.^32^ Recognising this potential tension, we consider that while information about each project’s policy on FoFs should be mentioned in consent documents and processes, such information should be in summary form. For instance, the consent form could state ‘no individual genetic research results will be given to you’, or mention the anticipatable findings that could be fed back to participants. It is important to note that volition alone is not sufficient for disclosure and that value and validity should be well articulated. It should also be made clear that participants also have a right to decline receiving their results.

#### Provide information about the choices people are asked to make if using tiered consent

For researchers using a broad consent model, it is important to point out that broad consent does not prevent researchers from providing feedback; absolve researchers from their ethical obligations, nor prevent them from recontacting participants. While it would be acceptable to use a tiered consent process, whereby participants are explicitly asked whether they would like to receive results and are given the possibility to opt out, there are also some challenges in tracking the different participant consents and where options for ‘tiered’ consent may not have been exhaustive.

Most importantly, where researchers choose to ask people for their preferences, they have to ensure that participants are appropriately informed about the potential information they may or may not receive and how that information may impact their lives.^10^ Where participants are not appropriately informed, the risk is that they would make decisions on the basis of partial information or misunderstanding, which could have real implications for their lives and well-being downstream. We recommend that engaging with participants on FoFs should start before the study commences, which is also an opportunity to explain and simplify further any difficult terminology or concept. Educational resources to support consent and feedback can be provided through community engagement activities or in conjunction with the H3Africa working group on Community Engagement. A study conducted by Torrorey-Sawe^33^ in Kenya piloted the use of an informed consent framework to implement FoFs for patients with breast cancer and their families. This could serve as a model and inform further research in other African settings.

### If we do decide to feedback, how should it be done?

#### Can verification be conducted in a laboratory that is certified/accredited for diagnostic testing?

In countries where genetic testing services are established (eg, USA, UK, South Africa), research identifies a participant who carries an actionable variant, the general practice is that those research results are then verified in an accredited diagnostic facility before any clinical action is taken. In the African research setting, clinical diagnostic verification of individual genetic research results in a clinical genetic diagnostic laboratory may be possible. However, one should also recognise the following challenges (1) not all tests are validated for use on the African continent, or indeed for all ethnic backgrounds, and, therefore, verification may require shipping samples overseas where facilities are appropriately licensed; this could prove to be prohibitively expensive and (2) capacity to ensure analytical validity (reproducibility) of the specific variant(s) may not yet be available and tests may still be in development, where these challenges are faced, and validation in a clinical genetic diagnostic laboratory is not feasible, it may be acceptable for researchers to *verify* relevant results using the resources readily available for the project. Verification could involve obtaining a second sample from the same person and rerunning the genomic analysis, possibly using more targeted genotyping or sequencing methods. Researchers should not feedback genetic research results that have not been verified, because mistakes—such as sample mix-up or mislabelling or experimental errors—do occur when handling and processing samples, or analysing data. Internal control single nucleotide polymorphism (SNP) genotyping is recommended to reduce the risk of inaccurate results due to mix-up and mislabelling of samples before transporting for sequencing.^34^

The decision on which approach to follow in order to verify genetic test results should be informed by the Standard of Care, policies and laws in the relevant country or countries. In countries where there is diagnostic clinical laboratory infrastructure, researchers should adhere to the standards and policies in those countries.

#### Who should do the feedback?

Internationally, there is a preference for individual genetic research findings to be fed back by medical genetic health professionals, preferably genetic counsellors or people with medical genetic training such as medical geneticists. There is a real shortage of health professionals with this kind of qualification in most African countries, meaning that they are probably not available to assist researchers in feeding back research results. H3Africa considers that the absence of genetic counsellors per se should not be a reason to preclude FoFs. Rather, the consortium should look for means to train other healthcare professionals in developing the essential skills required to communicate with participants about individual genetic research results. In the first instance, we consider it advisable that the task of feeding back information about individual genetic research results rests with clinicians and other qualified health professionals involved in the genomic research projects, until other staff are sufficiently trained to take over this task. In the study by Torrorey-Sawe^33^ a clinician was responsible for feeding back results with the support of the research team. Other healthcare staff that could take over these duties are, for example, psychologists, nurses, social workers or others who have been involved in the research process, have shown aptitude for communicating with participants about the research process and who are interested in a counselling role. It is also imperative to explore whether other methods may be appropriate for sharing results, such as telemedicine, which has shown great promise in expanding the availability of modern medical technologies to rural areas in Africa. However, this method of feedback should still be supported by a local clinician.

### Extending feedback of genetic results/findings to families

Genetic findings have implications for family members. Yet involving families in FoFs could violate a participant’s privacy and confidentiality. It is imperative that the privacy and confidentiality of the person enrolling in the study should be respected. In cases where there is benefit in sharing results with family members, the original participant should grant permission for them to be contacted. As with feedback to individuals, feedback should not be imposed on family members but should be based on their voluntary consent.

### Feedback of incidental findings

In the course of their research, investigators may encounter incidental findings (also called secondary findings)—clinically relevant genetic information about a research participant or patient that is identified outside the scope of the original research objective or diagnostic test being performed. These could be both anticipated and unanticipated. Where this is the case, it is important for researchers to understand ethical ways of handling these incidental findings.

As a result of the increased use of whole genome sequencing (WGS) methods, there is a likelihood of encountering incidental findings. The American College of Medical Genetics and Genomics (ACMG) provides guidelines for feedback of incidental findings^35 36^ However, as discussed above, clinical validity and relevance would still be a requirement for the target population. We recommend that the research team apply the same rigorous approach as provided herein for clinically valid and actionable results, but with an additional task of determination of relevance for the target population and should only be applicable for 1 and 2 above. However, due to the potential limited skills of the research team, it would be advisable to refer the participants to their doctor and for the process to follow the standard of care in that setting. It is important to note that as knowledge increases, the listed genes or variants may change.

## Conclusions and further recommendations

Considering that there is still a lot to learn about genetic variation in African populations, with a sparse evidence base about the preferences and understanding of research participants in the African continent, these guidelines will continue to evolve and be adapted as necessary. A next key step includes development of implementation guidelines for application in various settings, which will contribute to sharing best practices for FoFs in genomics research projects in Africa. To support these activities, there is an urgent need to develop in the following areas.

### Validation of clinical importance

- Establish an expert group that could be consulted by individual investigators trying to decide whether to feedback certain findings or not.
- Generate an evidence base for FoFs, where there is a sufficiently strong evidence base to support a decision to feedback in African populations.
- Build evidence on the clinical relevance of genes and variants in African populations. With an increasing generation of genomic sequence data and identification of novel variants in African genomes, there will be an increased need for reclassification of variants.^10 26 37^
- Engelbrecht *et al* 30 demonstrated the utility of next generation sequencing panels (NGS) panels in altering management in 67% of patients with inborn errors of immunity (EIE) in an African setting. Based on a clinical diagnostics environment, the low detection rate was due to high diversity and possibly undiscovered variants in African populations. However, the actionability was achieved in 67% of the diagnosed patients altering clinical management. The approach could be applied by African researchers and develop evidence base for their disease of study in their population contexts and clinical environments and could inform future needs and strategies for FoFs.

### Research and training

- Build an evidence base about how information about FoFs can best be integrated in the consent process and whether giving people a choice to receive results or not is meaningful and leads to informed choices. A study conducted in Kenya used an informed consent framework to inform implementation of FoFs among patients with breast cancer.^33^ The feedback process aligns closely with our guideline and decision tree framework. This serves as an ideal example of implementing FoFs and extending to families.
- There are few trained genetic counsellors in Africa, thereby presenting a clear need to develop a training platform to support effective task-shifting. These could be online modules aimed at clinicians in the first instance, and at other healthcare professionals subsequently and that would cover topics such as how to communicate risk and uncertainty. The absence of genetic counsellors in the Kenyan study by Torrorey-Sawe *et al* 33 meant that the feedback process was conducted by the local clinician who has understanding of the medical issues, and the socio-cultural factors, with expert support of the research team, a model which can also be adapted, where research and service delivery is feasible.
- Consider other factors which may determine strategies for FoFs, including stakeholder views and experiences on implementation of FoFs, context, age of participants, socioeconomic situation, disease conditions, demographics, funding resources and perceived outcomes of FoFs. Recent work by Mswaka *et al* 3 reported on community perceptions and individual preferences for the return of results in Uganda and a need to build national guidance to support genetic research. More localised research is critical to determine the best ways to implement FoFs in African settings.

## Decision flowchart

A decision flowchart is presented in figure 1, which provides a logical framework to assist in planning and decision-making on whether or not to provide FoFs.

### General points to consider for FoFs

- Develop feedback policy for projects, include in informed consent form (ICF), obtain IRB approval. Determine feedback on a *case by case* basis for a particular genetic finding.
- Consider specific details for single marker or group of markers or classical clinical genetics setup with monogenic traits versus complex with multiple risk factors.
- Determine the nature and possibility of (1) anticipated findings pertinent to disease or research question(s) and (2) anticipated incidental findings, which have a high probability to be found due to study demographics (eg, age, sex, disease status).
- For medical actionability, consider local specific issues (eg, availability of medicines, insurance).
- Costs of feeding back, logistics and timeframe for feedback should be addressed during the proposal stage.
- Determine analytical validity of methods used in confirming the results, that is, accuracy and reliability in detecting genetic variants in individuals.
- Genetic counselling should be done by a genetic counsellor or suitably qualified healthcare professional.
- Implications of findings on families—the same principles apply in evaluating value, obtaining consent and providing appropriate counselling.

## Summary points

We recommend the following general principles:

- The current approach should be to primarily feedback findings that are pertinent to the original research project. In that case, researchers need to
  - assess evidence base for potentially pathogenic variants in relation to the population(s) that are being investigated;
  - assess whether the particular finding likely holds value for the individual.
- Ensure that patients are appropriately informed of the implications of the findings for their disease or treatment and referred for follow-up care. Where these considerations are all fulfilled, then researchers should develop a feedback policy describing which findings they will feedback and when. The policy should be the same for the entire research project and all research sites.
- Where there is no national genetic diagnostic infrastructure, researchers must ensure that the research findings are verified before reporting back to ensure that participants are referred to appropriate care. Verification could involve obtaining a second sample from the same person, rerunning of the genomic test possibly using different methods (eg, low throughput/single marker genotyping and Sanger sequencing). Where there is a diagnostic genetic laboratory infrastructure, then researchers need to comply with the standards and regulations in that country.
- Information about the policy for FoFs for any specific project should be mentioned in consent documents and processes, but this could be in summary form. Where researchers opt to specifically get consent for feedback, then they need to ensure that participants are properly informed about the questions they have been asked and the implications of their choice.
- In the absence of genetic counsellors and until professional staff can be trained to meaningfully feedback individual genetic research results, we consider that the task of feeding back information about individual genetic research results rests with researcher–clinicians involved in the genomic research projects.
- Although heads of families and community leaders have an important influence in the decision-power over others in all aspects of their lives in Africa, and family feedback may be appropriate, the process of feeding back results should also have safeguards to ensure that the decision of the individual is met.
- In all cases, any decisions concerning the FoFs must be expressly approved by the research ethics board that governs the study and comply with all local and international regulations that govern such.

## Glossary

### Key definitions

The following definitions are important in context of this guideline.

- Analytical validity refers to the accuracy with which a particular genetic characteristic or marker is identified in a laboratory test.
- Clinical validity refers to the accuracy with which a test identifies a patient’s clinical status or disease condition.
- Clinical utility means that information from a genetic test can be used for informing effective management or prevention of a disease. An example of how clinical utility model was applied on a complex set of immune disorders—see Engelbrecht.^30^
- Complex (or multifactorial) diseases are due to effects from variations in several genes or loci and other factors such as environment and lifestyle.
- FoFs refer to the process of returning genetic results to individuals enrolled in a genetic/genomic research project.
- Genomic research: H3Africa projects are applying various analytical methods including candidate gene studies, whereby a set of markers or genes are investigated for association with specific disease traits. WGS, whole exome sequencing and genome-wide association studies enable interrogation of large genome regions generating large amounts of data. Analysis of these data produces findings, which undergo further assessments with the aim of identification of biomarkers, which are potentially useful for predicting disease risk, confirming diagnosis or guiding treatment and understanding human biology and disease mechanisms.
- Incidental findings are additional findings concerning a patient or research participant who may, or may not, have potential health implications and clinical significance, which are discovered during the course of a research study but are beyond the aims of the original test or investigation.
- Medical actionability: based on clinical validity and/or clinical utility and is defined as clinically prescribed interventions specific to the genetic disorder under consideration that are effective for prevention, delay of clinical disease or could lead to improved health outcomes. Examples include patient management (eg, risk-reducing surgery), surveillance (eg, colonoscopy) or specific circumstances or substances, which the patient should avoid (eg, certain types of anaesthesia).
- Monogenic disorder is caused by a variant in a single gene or locus.
- Pathogenicity: pathogenicity refers to the underlying measure of the extent to which the presence of a genetic variant is related to a particular disease or condition. Genotyping methods analyse known variants or candidate gene markers, and significant association in a cohort is used to confirm disease link, even when the function is unknown. Sequencing has the power to detect both known and novel variants, and prediction algorithms may be used to determine pathogenicity. Some of these variants could be predicted to be pathogenic but have no clinical utility. Web-based tools and software can be used for interpretation of sequence variants. The collaborative Clinical Genome Resource programme (https://www.clinicalgenome.org), funded by the National Human Genome Research Institute, provides well-curated databases and tools useful in the interpretation of clinical relevance of genes and variants. The Clinical Genome Resource Pathogenicity Calculator applies evidence-based reasoning to classify pathogenicity of sequence variants (http://calculator.clinicalgenome.org/site/cg-calculator).^38^ These tools apply standards and guidelines from the American College of Medical Genetics and Genomics - Association for Molecular Pathology (ACMG-AMP, USA) and the Association for Clinical Genomic Science (ACGS, UK).^36 39^ While these are more appropriate for Mendelian traits or for variants with large effects sizes, more rigorous approaches are required for most complex diseases, which are highly polygenic and multifactorial, with large numbers of underlying genetic variants, which individually have small effect sizes. Genetic risk scorescombine cumulative effect of multiple risk alleles to obtain a genetic risk score.
  - *Determination of pathogenicity and clinical validity in a research context:* an example of how to determine pathogenicity and clinical validity from new evidence for complex disorders, Garcia *et al* 22 provide a guide that may be useful, although with a focus on cardiomyopathies and arrhythmias. Similar approaches may be followed for a systematic evaluation of the pathogenicity of variants identified in clinically affected individuals, supported by multidisciplinary expert teams in the disease areas particularly for complex diseases.
- Pertinent findings: in a research context, findings are considered pertinent if generated or sought with the purpose of answering a particular clinical or research question either by genotyping specific areas of the genome or by specifically interrogating those areas if the whole genome has been sequenced.
- Personal utility: is the case where receiving information about the variant is important for individuals, for instance, because it ends a diagnostic odyssey, gives diagnostic closure, alerts to lifestyle-related risks or is important for reproductive health.

## Data Availability

All data relevant to the study are included in the article.
